# Development of late pulmonary hypertension after antenatal inflammation in experimental bronchopulmonary dysplasia

**DOI:** 10.1038/s41390-025-04223-6

**Published:** 2025-06-28

**Authors:** Paula Dias Maia, Gregory Seedorf, Tania Gonzalez, Elisa Bye, Benjamin S. Frank, Erica W. Mandell, Steven H. Abman

**Affiliations:** 1 Section of Neonatology, Department of Pediatrics, University of Colorado Anschutz School of Medicine and Children’s Hospital Colorado, Aurora, CO USA; 2 Pediatric Heart Lung Center, Department of Pediatrics, University of Colorado Anschutz School of Medicine and Children’s Hospital Colorado, Aurora, CO USA; 3 Section of Cardiology, Department of Pediatrics, University of Colorado Anschutz School of Medicine and Children’s Hospital Colorado, Aurora, CO USA; 4 Section of Pulmonary Medicine, Department of Pediatrics, University of Colorado Anschutz School of Medicine and Children’s Hospital Colorado, Aurora, CO USA

## Abstract

**Background:**

Antenatal inflammation due to chorioamnionitis is strongly associated with the development of bronchopulmonary dysplasia (BPD) and BPD-associated pulmonary hypertension (BPD-PH) after preterm birth. However, mechanisms linking antenatal stress with temporal changes in the pulmonary circulation leading to BPD-PH during infancy are incompletely understood. We hypothesized that antenatal inflammation impairs lung alveolar and vascular growth that precedes and increases susceptibility for the development of late BPD-PH.

**Methods:**

Fetal rats received intra-amniotic ETX or saline 2 days before term delivery. We quantified temporal changes in lung structure, mechanics, and pulmonary hemodynamics by echocardiogram at postnatal days 2 (P2), P7, and P14.

**Results:**

At P2, ETX-exposed pups showed decreased alveolar and vascular growth, without evidence of right ventricular hypertrophy (RVH). Though echocardiography revealed increased interventricular septal flattening, other markers of PH were not different between groups. By P7–P14, echo revealed changes in metrics of PH and RVH. We found sustained reduction of pulmonary vascular and alveolar growth, increased pulmonary artery wall thickness, and worsened lung mechanics in ETX-exposed pups during infancy.

**Conclusions:**

Antenatal inflammation impairs lung vascular growth shortly after birth and causes early pulmonary vascular disease, which precedes sequential changes in pulmonary artery remodeling and echocardiogram markers of PH during the postnatal period, even in the absence of postnatal injury.

**Impact:**

- No published preclinical studies have investigated the association between antenatal inflammation (AI) and temporal changes in structure and function of the developing lung circulation that lead to pulmonary hypertension (PH) associated with bronchopulmonary dysplasia (BPD; BPD-PH), despite strong epidemiologic links.
- We demonstrate that AI causes early abnormalities of pulmonary alveolar and vascular growth, preceding changes in pulmonary mechanics and echocardiographic metrics of PH, which increase over time during infancy without adverse postnatal exposures.
- These findings support the growing evidence that AI disrupts lung vascular development and that early pulmonary vascular disease increases susceptibility to BPD and BPD-PH in infancy.

## Introduction

Remarkable cardiorespiratory advances in perinatal and neonatal care in recent decades have led to increased survival of the most extremely premature newborns at the limits of viability, including those at 22–23 weeks’ gestation. While these significant strides have reduced many postnatal complications, the incidence of bronchopulmonary dysplasia (BPD), the chronic lung disease of prematurity, has not changed over the past decades and remains the most common prematurity-related mortality and co-morbidity, leading to long-term cardiorespiratory sequelae across the lifespan.^1,2^ Among preterm infants who develop BPD, nearly one-third of surviving infants develop severe BPD and are at especially high risk for death, prolonged ventilator and oxygen support, pulmonary hypertension (PH), recurrent respiratory exacerbations, repeated hospitalizations, exercise intolerance, and late neuro-developmental sequelae.^3^

Among many comorbidities, the late development of BPD-associated PH (BPD-PH) is strongly linked with mortality and poor outcomes.^4^ Premature birth has been recognized as strongly associated with early PH immediately after birth, which includes severe persistent PH of the newborn, delayed pulmonary vascular transition, or high flow due to a large patent ductus arteriosus.^5,6^ While some preterm infants experience resolution of their early or acute postnatal PH, others are particularly at risk for developing late and chronic pulmonary vascular disease (PVD), even in the absence of significant PH during the acute transitional period.^6^ As most recently highlighted in the Seventh World Symposium of Pulmonary Hypertension,^7^ the late development of BPD-PH after preterm birth can present later during the Neonatal Intensive Care Unit course and persist into infancy, childhood, and young adulthood and may contribute to adult-onset PH. This temporal pattern represents a distinct clinical course and physiology from other phenotypes of early PVD in preterm infants.^6,8^ Recognition of this high risk for late PH led to several consensus recommendations from the American Heart Association, the American Thoracic Society, and the Pediatric Pulmonary Hypertension Network to recommend screening for PH by echocardiogram in preterm infants with BPD at 36 weeks as well as during long-term follow-up.^8,9^ However, the optimal timing of diagnosis and management of BPD-PH remain a major clinical challenge, and mechanisms linking perinatal factors with late PH are uncertain.^4^

Despite growing appreciation for the significant contribution of antenatal factors, including chorioamnionitis and placental dysfunction^3,10–12^ with early PVD as precursors of BPD-PH,^13–17^ uncertainty exists regarding the timing, progression, and mechanisms underlying the development of late PH following antenatal stress. Past preclinical studies have clearly demonstrated the presence of impaired lung vascular growth and PH in experimental models of PH,^18^ and have further shown that disruption of angiogenesis further increases risk for impaired alveolarization, contributing to the pathogenesis of BPD as well as BPD-PH (the “vascular hypothesis of BPD”).^19,20^

In addition, strong epidemiologic and clinical data have clearly implicated the role of adverse antenatal determinants such as chorioamnionitis, maternal smoking, and placental dysfunction, with high risk for BPD-PH.^3,10–12^ Importantly, early echocardiographic evidence of even mild PVD at 7 days of age has been associated with high risk for subsequently developing BPD and late BPD-PH.^14^ However, precise temporal changes in lung vascular growth, structure, and pulmonary mechanics as well as mechanisms linking antenatal stress with late PH in preterm birth are incompletely understood.

Therefore, we hypothesized that antenatal inflammation causes early disruption of lung alveolar and vascular growth that precedes and increases susceptibility for the development of late PH in chorioamnionitis-induced BPD. To test this hypothesis and better understand the temporal changes associated with late PH in the context of BPD, we studied longitudinal changes in lung structure and pulmonary mechanics after exposure to antenatal inflammation in a well-established rodent model of chorioamnionitis-induced BPD with serial assessments of distal lung airspace and vascular development, lung mechanics, transthoracic echocardiographic measurements of PH and right ventricular hypertrophy (RVH), and direct assessment of RVH.

## Materials and methods

**Fig. 1:**
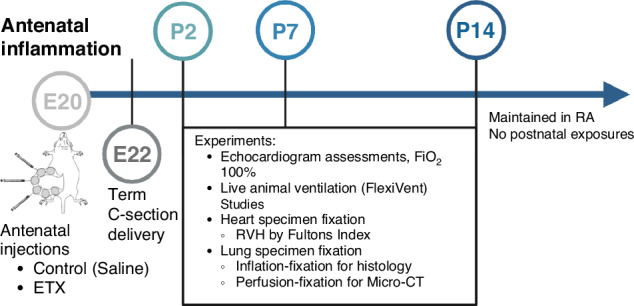
Schematic illustration of the experimental model and study design. C-section cesarean section, P2 postnatal day 2, P7 postnatal day 7, P14 postnatal day 14, E20 embryonic day 20, E22 embryonic day 22, ETX endotoxin, FiO_2_ fraction of inspired oxygen, RA room air, RVH right ventricular hypertrophy. *n* = 7–12 animals per group.

Animal procedures were approved by the University of Colorado Denver Institutional Animal Care and Use Committee (Protocol #00339, AAALAC #00235). The experimental study design is illustrated in Fig. 1.

### Experimental chorioamnionitis

Intra-amniotic saline (CTL) injections or 10 µg lipopolysaccharide endotoxin (ETX) injections of embryonic day 20 (E20) rat fetuses were performed as previously described.^21–23^ All experimental endpoints were obtained at postnatal day 2 (P2) when rat lungs are at the saccular stage of lung development and at days 7 (P7) and 14 (P14), during the early alveolar stage in rodents.^24^ *N* = 7–12 animals per experimental group.

### Transthoracic echocardiogram

Supine-positioned animals on the warmed stage of the echocardiogram system were anesthetized with inhaled isoflurane (1–3%) and 100% FiO_2_ via nosecone while spontaneously breathing, titrated to maintain heart rates at 250–300 beats/min and body temperature between 37.5 and 37.8 °C. Chest hair was removed using depilatory cream. Serial echocardiograms were performed in CTL and ETX-exposed rat pups at P2, P7, and P14 to assess serial changes in pulmonary hemodynamics by multiple metrics, including left ventricular end-systolic eccentricity index (LVEI), right ventricular wall thickness at diastole (RVWTd), pulmonary artery acceleration time to ejection time ratio (PAAT/PAET), and right ventricular systolic to diastolic duration ratio (RV S:D).

Pulmonary hemodynamics and RV morphology were assessed by two-dimensional transthoracic echocardiography acquired from the modified parasternal long-axis and short-axis views using B-mode, M-mode, color Doppler, and pulse wave Doppler using a Visual Sonics Vevo 1100 ultrasound system (Visual Sonics, Toronto, Ontario, Canada) and a 13–24 MHz linear transducer (MS-250) MicroScan solid-state transducer and analyzed as previously described.^25–27^

The interventricular septal configuration was evaluated through B-mode images from a modified midpapillary left parasternal short-axis view. LVEI was calculated as the ratio of the LV anterior-inferior to the septo-lateral diameters.

RV morphology was assessed with RVWTd by M-mode echocardiography from a modified right parasternal long-axis view.^25^ The pulmonary artery (PA) was visualized using B-mode from a modified left parasternal long-axis view. The optimal window for PA flow measurements was identified using color Doppler at the crossing point between the proximal aorta and PA, followed by pulse-wave Doppler to assess the PA flow time intervals. PAAT and PAET were measured from the onset of systolic ejection to peak flow velocity and the onset of PA ejection to the point of systolic pulmonary arterial flow cessation, respectively. To account for heart rate variability, PAAT was adjusted to ejection time and presented as PAAT/PAET. To calculate RV S: D, we used the ratio between PAET and the total duration of the cardiac cycle minus the PAET. All measurements were performed offline by one blinded investigator over three consecutive heartbeats, and values were expressed as the mean ± SEM.

### Mechanical ventilation

Total respiratory system resistance and total lung compliance were measured on small animal ventilators (Flexivent FX1, FX2; SCIREQ, Montreal, Canada) as previously described.^28^

### Distal lung morphometrics analysis of inflation-fixed lungs

Lungs were inflation-fixed in situ, as previously described by our laboratory,^21^ with alveolarization assessed via radial alveolar counts (RAC) and mean linear intercept (MLI).^28–30^ After staining with von Willebrand factor, an endothelial cell-specific marker, pulmonary vessel density, and pulmonary vascular wall thickness were assessed as previously described by our laboratory.^21^

### Micro-lung CT and image analysis

Ex vivo imaging of whole lung specimens was performed for pulmonary arterial vessel comparison analysis. Lungs were perfusion-fixed using a method previously established as representative of the in vivo murine lung.^31^ Micro-computed tomography (micro-CT) images were obtained with a Quantum GX2 micro-CT Imaging System (PerkinElmer, Waltham, MA) and analyzed using 3D Slicer (Slicer, v.4.11).

### RVH

Hearts were dissected and weighed at each experimental time point. The right ventricle (RV) and left ventricle plus septum (LV + S) were dissected and weighted, and the ratio of RV to LV + S weights (Fulton’s Index)^21,23^ was determined.

### Verification of animal sex

Animal sex was determined by polymerase chain reaction from DNA isolated from rat tail clips using Extract-N-Amp (XNAT2, Sigma Aldrich) and visualized by agarose gel electrophoresis as previously described.^22^

### Statistical analysis

Data are presented as the mean ± SD. Statistical analysis was performed using GraphPad Prism (v.10.3.0, GraphPad Software, La Jolla, CA). Data were then analyzed using the Mann–Whitney *T* test and Kruskal–Wallis analysis of variance for multiple comparisons. A *p* value of <0.05 (*) was considered statistically significant.

## Results

### Morphometric assessments of serial changes in distal lung structure

**Fig. 2:**
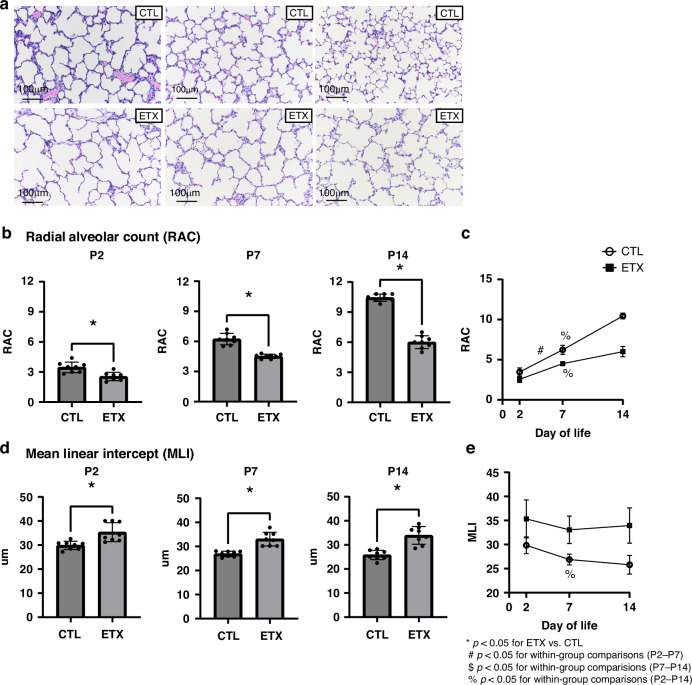
Effects of antenatal ETX exposure on distal lung structural development at P2, P7, and P14. **a** Representative lung histology images demonstrate alveolar simplification in EXT-exposed rat pups compared to saline controls (CTL); magnification ×20; scale bar 100 μm. **b**, **d** Morphometric analysis of inflation-fixed lung tissue shows that antenatal ETX exposure leads to distal lung simplification quantified by decreased radial alveolar count (RAC, **b**) and increased mean linear intercept (MLI, **d**). **c**, **e** Temporal changes in RAC and MLI over time. While biologically relevant trends are observed, most within-group comparisons across time points did not reach statistical significance, except for P2–P7 and P2–P14 in the CTL group and P2–P14 in the ETX group (RAC, **c**), and P2–P14 in the CTL group (MLI, **e**). *p < 0.05 for ETX vs. CTL; ^#^*p* < 0.05 for P2–P7 within-group comparison; ^$^*p* < 0.05 for P7–P14 within-group comparison; ^%^*p* < 0.05 for P2–14 within-group comparison. *n* = 8 animals per group.

Histology-based comparisons of inflation-fixed distal lung tissue of CTL and antenatal ETX-exposed pups revealed early impaired lung growth after birth, as evidenced by increased distal airspaces at P2, which corresponds to the saccular stage of lung development, and persisted at P7 and P14, which correspond to the early and late alveolar stages, respectively. Compared to CTL rats, lungs from antenatal ETX-exposed infant rats show a 27% difference in RAC at P2 and at P7, and a 43% decrease by P14 (*p* < 0.05 for each comparison). Similarly, MLI significantly increased in ETX-exposed lungs by 22% at P2, 23% at P7, and 31% at P14 (*p* < 0.05 for each comparison), further supporting persistent abnormalities in distal airspace or alveolar structure (Fig. 2).

### Effects of antenatal ETX on pulmonary vascular growth and structure

**Fig. 3:**
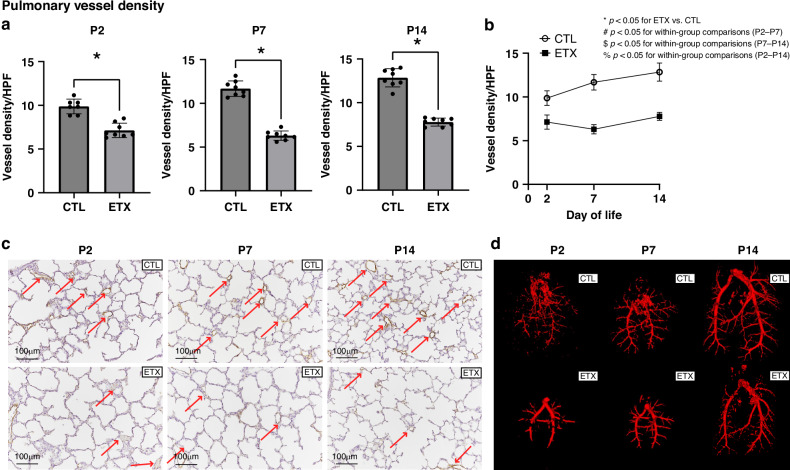
Effects of antenatal ETX exposure on pulmonary vascular development at P2, P7, and P14. **a** Quantitative analysis demonstrates that ETX-exposed pups exhibit significantly reduced pulmonary vessel density at all time points compared to saline controls (CTL), with no significant increase in vessel density over time. **b** Temporal changes in pulmonary vessel density. While biologically relevant trends are observed, within-group comparisons across time points did not reach statistical significance. **c** Representative lung histology images showing pulmonary vessels (red arrows). ETX-exposed pups demonstrate decreased vascular density compared to CTL; magnification ×20; scale bar = 100 μm. **d** Lung micro-CT angiograms following barium vascular injection illustrate reduced vascular filling and distal branching in ETX-exposed pups compared to CTL at all time points. *p < 0.05 for ETX vs. CTL; ^#^*p* < 0.05 for P2–P7 within-group comparison; ^$^*p* < 0.05 for P7–P14 within-group comparison; ^%^*p* < 0.05 for P2–14 within-group comparison. *n* = 7–8 animals per group.

Lung histology demonstrated that ETX-exposed infant rats had decreased pulmonary vascular density when compared with controls (CTL) at P2, P7, and P14. In comparison with CTL pups, antenatal ETX-exposed pups had lower pulmonary vessel density at P2 by 31.5%, which persisted at P7 by 46.2%, and at P14 by 38.6% (*p* < 0.05 for each comparison). Lung micro-CT and angiograms after barium vascular injection illustrated decreased filling and reduced distal branching in the pulmonary vasculature of ETX-exposed infant rats at P2, P7, and P14 compared to CTL rats (Fig. 3).

**Fig. 4:**
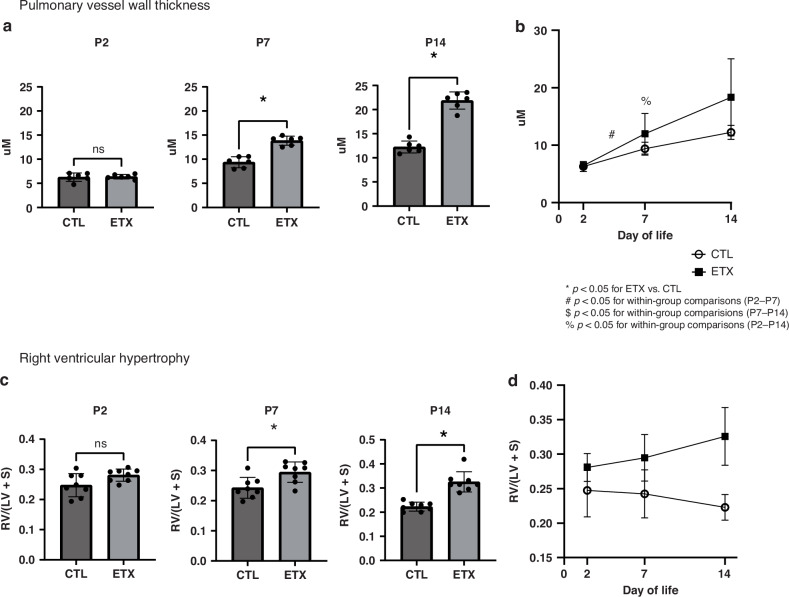
Effects of antenatal ETX exposure on pulmonary vascular wall thickness and right ventricular hypertrophy (RVH) at P2, P7, and P14. **a** No difference in pulmonary vessel wall thickness was observed at P2. However, ETX-exposed pups exhibited increased pulmonary vessel wall thickness at P7 and P14, suggesting pulmonary arterial remodeling. **b** Temporal changes in pulmonary artery wall thickness. While biologically relevant trends are observed, most within-group comparisons across time points did not reach statistical significance, except for P2–P7 and P2–P14 in the ETX group. **c** No difference in RVH, assessed by Fulton’s Index, was observed between the CTL and ETX groups at P2, but ETX-exposed pups demonstrated increased RVH at P7 and P14. **d** Changes in RVH over time. While biologically relevant trends are observed, within-group comparisons across time points did not reach statistical significance. *p < 0.05 for ETX vs. CTL; ^#^*p* < 0.05 for P2–P7 within-group comparison; ^$^*p* < 0.05 for P7–P14 within-group comparison; ^%^*p* < 0.05 for P2–14 within-group comparison; ns not significant. *n* = 6–8 animals per group. LV + S left ventricle plus septum, RV right ventricle.

In addition to studies of pulmonary vascular density, we further quantified small PA remodeling at each time point. There was no difference in small PA wall thickness between CTL and ETX-exposed study groups at P2. However, ETX-exposed pups exhibited a significant increase in vessel wall thickness at P7 (by 48.5%) and D14 (by 85.5%) compared to CTL pups (*p* < 0.05 for each comparison), demonstrating progressive changes in small PA wall thickness in this model. We further found that RVH as assessed by Fulton’s index (RV/LV + S ratio) was not different between the CTL and ETX groups at P2. However, ETX-exposed pups had evidence of increased RVH by 31% at P7 and by 40.4% at P14 compared to CTL pups (*p* < 0.05 for each comparison) (Fig. 4).

### Effects of antenatal ETX on lung mechanics throughout infancy

**Fig. 5:**
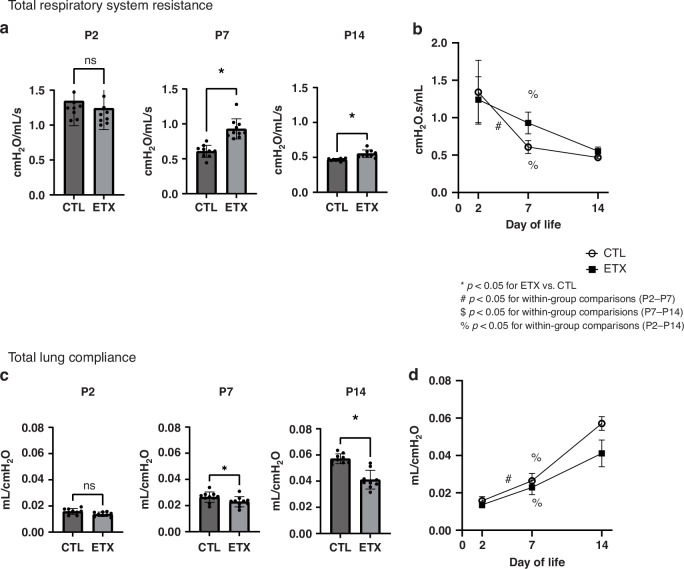
Effects of antenatal ETX exposure on lung mechanics at P2, P7, and P14. **a** While no significant difference was observed at P2, ETX-exposed pups exhibited increased total respiratory system resistance at P7 and P14 compared to controls. **b** Temporal changes in total respiratory system resistance. While biologically relevant trends are observed, most within-group comparisons across time points did not reach statistical significance, except for P2–P7 and P2–P14 in the CTL group and P2–P14 in the ETX group. **c** ETX-exposed pups demonstrated reduced total lung compliance at P7 and P14, despite no significant difference at P2 compared to controls. **d** Temporal changes in total lung compliance. While biologically relevant trends are observed, most within-group comparisons across time points did not reach statistical significance, except for P2–P7 and P2–P14 in the CTL group and P2–P14 in the ETX group. *p < 0.05 for ETX vs. CTL; ^#^*p* < 0.05 for P2–P7 within-group comparison; ^$^*p* < 0.05 for P7–P14 within-group comparison; ^%^*p* < 0.05 for P2–14 within-group comparison; ns not significant. *n* = 9–10 animals per group.

Although not different at P2, ETX-exposed rat pups showed an increased total respiratory system resistance when compared to that in CTL pups at P7 by 38.6% and at P14 by 15.7% (*p* < 0.05 for each time point). Total lung compliance at P2 was similar between ETX-exposed and CTL pups. However, the ETX-exposed pups had decreased total lung compliance at P7 by 13% and at P14 by 31% when compared to that in CTL pups (*p* < 0.05 for each comparison) (Fig. 5).

### Effects of antenatal ETX on pulmonary hemodynamics and RV wall thickness as assessed by transthoracic echocardiogram throughout infancy

#### LVEI

**Fig. 6:**
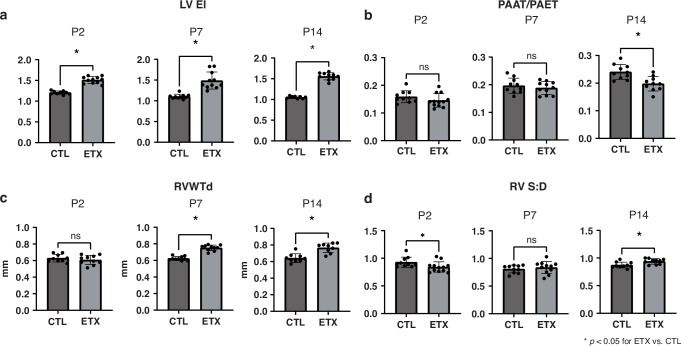
Effects of antenatal ETX exposure on pulmonary hemodynamics and right ventricular (RV) wall thickness and function at P2, P7, and P14, as assessed by transthoracic echocardiography. **a** ETX-exposed pups exhibited a significant increase in the left ventricular end-systolic eccentricity index (LVEI) at P2, P7, and P14 compared to control (CTL) pups. **b** No significant difference in right ventricular wall thickness at diastole (RVWTd) was observed between CTL and ETX groups at P2. However, ETX-exposed pups had increased RVWTd at P7 and P14 compared to CTL pups. **c** Pulmonary artery acceleration time to ejection time ratio (PAAT/PAET) did not differ between CTL and ETX-exposed pups at P2 and P7, but was significantly decreased in ETX-exposed pups at P14. **d** Right ventricular systolic-to-diastolic duration ratio (RV S:D) was significantly decreased in ETX-exposed pups at P2, not different at P7, and increased at P14 compared to CTL *p < 0.05 for ETX vs. CTL; ns not significant. *n* = 8–12 animals per group.

**Fig. 7:**
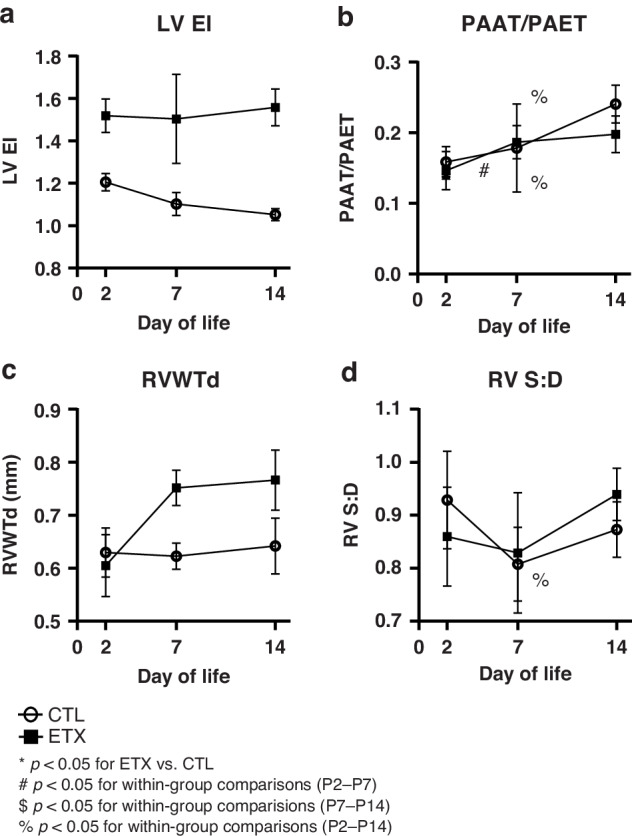
Temporal changes in pulmonary hemodynamics and right ventricular (RV) wall thickness and function following antenatal ETX exposure, as assessed by transthoracic echocardiography. **a** Left ventricular end-systolic eccentricity index (LVEI). **b** Right ventricular wall thickness at diastole (RVWTd). **c** Pulmonary artery acceleration time to ejection time ratio (PAAT/PAET). **d** Right ventricular systolic-to-diastolic duration ratio (RV S:D). While biologically relevant trends were observed, most within-group comparisons across time points did not reach statistical significance, except for P2–P14 in the CTL group (PAAT/PAET), P2–P7 and P2–P14 in the ETX group (PAAT/PAET), and P2–P14 in the ETX group (RV S:D). *p < 0.05 for ETX vs. CTL; ^#^*p* < 0.05 for P2–P7 within-group comparison; ^$^*p* < 0.05 for P7–P14 within-group comparison; ^%^*p* < 0.05 for P2–14 within-group comparison. *n* = 8–12 animals per group.

Compared to CTL pups, ETX-exposed pups exhibited a significant increase in LVEI at P2 (control: 1.20 ± 0.04 vs. ETX: 1.51 ± 0.08; 25.2% difference; *p* < 0.05), suggesting increased RV systolic pressure. This difference between study groups was present at P2, persisting at P7 (control: 1.10 ± 0.05 vs. ETX: 1.48 ± 0.20; a 351% difference; *p* < 0.05) and P14 (control: 1.05 ± 0.02 vs. ETX: 1.55 ± 0.08; a 48.1% difference; *p* < 0.05) (Figs. 6 and 7).

#### RVWTd

There was no difference in RVWTd between the CTL and ETX groups at P2. However, RVWTd was increased in ETX-exposed pups at P7 (control: 0.62 ± 0.02 mm vs. ETX: 0.75 ± 0.03 mm; 20.68% difference; *p* < 0.05) and P14 (control: 0.64 ± 0.05 mm vs. ETX: 0.76 ± 0.05 mm; 19.35% difference; *p* < 0.05) (Figs. 6 and 7).

#### PAAT/PAET

PAAT/PAET ratios were not different between CTL and ETX-exposed pups at P2 and P7. However, at P14, ETX-exposed pups showed a significant decrease in PAAT/PAET compared to CTL pups (control: 0.24 ± 0.02 vs. ETX: 0.19 ± 0.02; a 17.8% difference, *p* < 0.05), suggesting increased pulmonary vascular resistance (Figs. 6 and 7).

#### RV S:D

Compared to the CTL group, RV S:D was decreased in ETX-exposed pups at P2 (control: 0.92 ± 0.09 vs. ETX: 0.84 ± 0.09; a 9.3% difference, *p* < 0.05), suggesting a potential RV hyperdynamic state. In comparison with CTLs, RV S:D was not significantly different at P7 in ETX exposed rats but was increased at P14 (control: 0.87 ± 0.05 g vs. ETX: 0.94 ± 0.04; 7.9% difference, *p* < 0.05), indicating signs of development of RV maladaptation and global dysfunction (Figs. 6 and 7).

Additional data analysis showed no significant differences in body weight, crown-rump length, and survival rates within the study groups. Furthermore, we found no differences in the proportion of male to female pups at any of the study time points (P2 CTL: 40% male vs. 60% female, P2 ETX: 50% male vs. 50% female, P7 CTL: 40% male vs. 60% female, P7 ETX: 50% male vs. 50% female, P14 CTL:60% male vs. 40% female P14 ETX: 70% male vs. 30% female). There were no sex differences in any study endpoints throughout the study; however, this study was not powered to sufficiently determine potential differences based on sex.

## Discussion

**Fig. 8:**
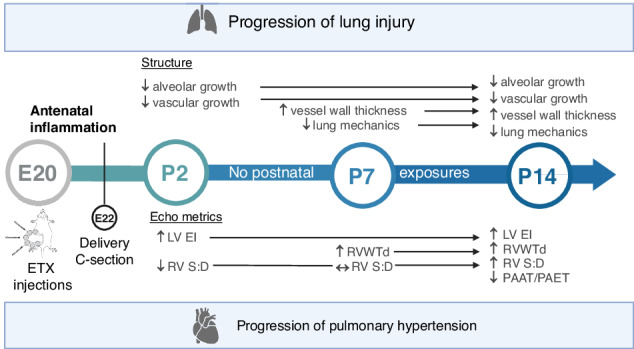
Effects of antenatal ETX on cardiopulmonary disease. Exposure to antenatal ETX caused early and sustained abnormalities of pulmonary alveolar and vascular growth that preceded changes in pulmonary mechanics and echocardiographic metrics of pulmonary hypertension, which increased over time throughout the first 2 weeks of life despite the absence of adverse postnatal exposures. C-section cesarean section, P14 postnatal day 14, P2 postnatal day 2, P7 postnatal day 7, E20 embryonic day 20, E22 embryonic day 22, ETX endotoxin, LVEI left ventricular end-systolic eccentricity index, PAAT/PAET pulmonary artery acceleration time to ejection time ratio, RV S:D right ventricular systolic to diastolic duration ratio, RVWTd right ventricular wall thickness at diastole.

Recent studies have identified distinct physiologic phenotypes of PVD in preterm infants, which include distinguishable patterns of the onset of early and late PH after birth.^6,7^ The presence of late PH is associated with high mortality and morbidity in preterm infants with BPD.^4,16,17,32^ However, the mechanisms underlying the timing of longitudinal changes leading to the subsequent development of BPD-PH are poorly understood. Using an established antenatal inflammation model of BPD to mimic chorioamnionitis, we demonstrated that exposure to antenatal ETX caused early and sustained abnormalities of pulmonary alveolar and vascular growth that preceded changes in echocardiographic metrics of PH throughout the first two weeks of life. We further found that antenatal ETX exposure impaired postnatal distal lung structure, during the saccular and alveolar periods, as well as abnormal lung mechanics with reduced total respiratory system compliance and increased resistance throughout the first 14 days of life. Additionally, antenatal inflammation disrupted pulmonary vascular growth, which was evident as early as the second day after birth. Changes in vessel density were followed by structural changes in PA wall remodeling and correlated with worsening of pulmonary hemodynamics during infancy (Fig. 8).

These findings are interesting as they suggest that antenatal stress causes early changes in lung vascular development with PVD, which precedes the development of late PH, even in the absence of postnatal injury. Additionally, we highlight the complexity of pulmonary vascular development following antenatal inflammation (Fig. 3) that may not be captured by two-dimensional histology and anticipate future studies incorporating advanced quantitative imaging methodologies will further define this complexity.

Recent clinical studies have shown that the early identification of PVD by echocardiogram at postnatal day 7 in human preterm infants is strongly associated with the subsequent development of BPD and BPD-PH.^13–17^ The results of this current study are the first to demonstrate the sequence of longitudinal progression of changes in lung vascular structure and function, as early PVD evolves into progressive changes reflecting overt BPD-PH in an animal model. These findings contribute to the growing body of literature evidencing the critical role of antenatal inflammation in the pathogenesis of early PVD, which increases susceptibility for both BPD and BPD-PH.^10,11^ Uncovering the mechanisms driving late PH development and progression has significant implications for more precise diagnostic assessment and clinical care and will help shift the current clinical BPD treatment paradigm, focused on postnatal therapies, towards interventions that provide early identification of at-risk neonates and enable more precise therapeutic strategies.^4^

Since its original description by Northway and colleagues over 50 years ago,^33^ substantial evidence has supported the understanding that while disease severity is definitely modulated by the adverse effects of postnatal exposures, antenatal risk factors, including chorioamnionitis^3,8–10^ and the presence of early PVD, are key contributors to the persistent BPD incidence and increased risk for BPD-PH.^3,4^ The pathophysiology of BPD-PH is multifactorial, with complex interactions between antenatal and postnatal exposures and stresses modulating disease risk, severity, and the development of distinct cardiopulmonary phenotypes and clinical trajectories of BPD-PH.^3,4^ However, strong clinical data demonstrate strong associations between antenatal stress and placental dysfunction with increased risk for developing BPD and BPD-PH.^11^ Our findings are supported by strong epidemiological and preclinical studies that have identified important roles for antenatal factors, which serve as major determinants of risk for BPD, BPD-PH, and late outcomes even in the absence of postnatal injuries due to such factors as hyperoxia, invasive ventilation, sepsis, and other stresses, which are also well-established contributors.^3,10–12^ Among those antenatal factors, chorioamnionitis with antenatal inflammation has been strongly associated with the risk for BPD and BPD-PH, especially when fetal growth restriction, as a biomarker for severe placental dysfunction and fetal stress, is present.^10–12,34^

In addition, recent key prospective clinical studies have shown that early evidence of PVD, evaluated and identified by echocardiogram in the first 7–10 days after birth, is associated with the development of BPD and BPD-PH at 36 weeks post-menstrual age (PMA) and increased mortality.^13–17^ These clinical findings are supported by important pre-clinical work from our laboratory that led to the development of the “vascular hypothesis” of BPD.^19,20^ These studies revealed that selective inhibition of angiogenesis and impairment of pulmonary vascular development disrupt alveolarization and cause BPD and PH in infant rats through disruption of endothelial–epithelial “angiocrine signaling,” which causes abnormal endothelial–epithelial communication and decreased airspace growth.^19,20^ In addition, other relevant and more recent pre-clinical studies suggest that the disruption of angiogenesis by adverse antenatal factors, such as chorioamnionitis, preeclampsia, fetal growth restriction, or maternal smoking, can cause PVD that not only leads to PH but can also impair distal lung growth.^10,32^

In this well-established rat model, our laboratory has previously shown that a single dose of antenatal ETX (delivered via injection into the amniotic fluid) administered during the late canalicular stage of lung development, in the absence of any postnatal injury, is sufficient to cause pronounced lung inflammation, stunt distal lung development with alveolar simplification, decrease pulmonary vasculature, alter lung mechanics, and cause RVH at 2 weeks of life, closely mimicking features observed in human BPD.^23,35^ We additionally demonstrated increased RV afterload and impaired RV-PA coupling by direct tissue assessment and cardiac catheterization at 2 weeks of age in the same model.^36^ Building on this existing knowledge, our novel findings on the timing and progression of airspace and pulmonary vascular growth impairment that led to late PH have potential direct translational applications, as future research can target specific therapeutic windows that could be used in neonatal populations to mitigate BPD and late respiratory outcomes in the most vulnerable preterm infants.

Echocardiography is commonly used in clinical BPD-PH, as it has been demonstrated to be a reasonably sensitive, non-invasive method for the identification and longitudinal follow-up of PH in infants with BPD.^4,8,37^ However, in the absence of a tricuspid regurgitation jet, which is only detected in 61% of the infants with BPD-PH or post-tricuspid shunt lesions, the reliability of quantifying mean pulmonary arterial pressure (PAP) by echocardiography decreases, and the qualitative and quantitative assessment of PH should be multiparametric.^38,39^ Although in experimental PH, cardiac catheterization and histopathologic studies to characterize PH are invasive terminal procedures that require a large number of subjects for longitudinal studies, only a few studies have used echocardiography to assess PH and related hemodynamics.^25^

Our study is the first to describe in a rodent model the temporal changes in the development of PH, as evidenced by multiple echocardiographic parameters in the absence of postnatal injury during the first 2 weeks of life following antenatal exposure to ETX. We demonstrated increased LVEI, suggesting increased RV systolic pressure, traditionally accepted as a surrogate to PAP, as early as P2. By P7, we found evidence of alteration in RV morphology (hypertrophy) by echocardiogram (RVWTd) and Fulton’s index. By 2 weeks of life, several echocardiographic metrics assessed were abnormal, including decreased PAAT/PAET, which is inversely correlated with pulmonary vascular resistance and PAP, and increased RV S:D. At P2, ETX-exposed pups exhibited a shortened RV systolic duration compared to controls, suggesting an early hyperdynamic RV state likely due to systemic inflammation causing myocardial stress. This early response contrasts with later time points (P7–P14), where RV S:D increases, suggesting RV maladaptation and global dysfunction in the setting of PH,^40–43^ while the CTL group followed a normal postnatal decline in RVH over time.

Based on our findings, we speculate that echocardiography is a valuable noninvasive tool to longitudinally study the temporal progression of cardiopulmonary disease in experimental BPD-PH and that the echocardiographic metrics used in this study are feasible in animal models of BPD-PH. Although the utility of specific echocardiographic parameters in BPD-PH assessment remains uncertain, our study suggests that LVEI may serve as an early and sensitive marker of abnormal RV pressure load due to PVD over time that leads to late PH. Supporting this, a prospective clinical cohort study evaluating the strength of associations between echocardiographic measurements of RV mechanics and BPD severity in a cohort of preterm infants at 36 weeks PMA^44^ found that LVEI was the only parameter associated with BPD severity and may be a useful tool to characterize RV mechanics in patients with BPD.^13,16,39^. Further echocardiographic studies in experimental BPD-PH may continue to provide valuable insights into using LVEI and other metrics for screening, longitudinal assessment of disease, and monitoring the effect of different management strategies in BPD-PH. Furthermore, additional investigations will continue to explore the pulmonary and systemic circulations in this model using cardiac catheterization.

Future studies are needed to continue to explore the mechanisms underlying disease progression and further link our findings with novel signaling pathways and inflammatory mediators underlying these changes. Previous preclinical studies suggest that disruptions in angiogenesis due to antenatal factors—such as chorioamnionitis, preeclampsia, and maternal smoking—and postnatal injury after preterm birth can cause PVD, which not only leads to BPD-PH but also impairs distal lung development. Preclinical research has also demonstrated that the developing pulmonary endothelial cells are crucial in regulating and coordinating epithelial growth and the structure of distal airspaces through the production of critical “angiocrines” including nitric oxide (NO), hepatocyte growth factor, vitamin A, insulin-like growth factor-1, and others.^19,23,45,46^ Since angiogenesis is essential for normal alveolarization,^19,20,47^ it has been proposed that protecting the developing pulmonary vasculature from early injury may not only lower the risk of PH and improve gas exchange but also enhance distal lung growth and improve long-term respiratory outcomes.^48^ Upcoming studies will investigate the mechanisms underlying these findings and examine changes in pulmonary endothelial cell heterogeneity and function, as well as novel pathways and inflammatory mediators, that may contribute to the impairment of lung vascular development.

This study has several potential limitations. Echocardiograms were performed while the animals were sedated but spontaneously breathing high FiO_2_ to avoid the acute vasoconstrictor effects of acute hypoxia in order to standardize study conditions. Future studies exploring the role of altered pulmonary vasoreactivity in addition to the vascular growth and structural changes found in this study would be of marked interest. Nevertheless, this study offers valuable insights into how antenatal inflammation alters pulmonary hemodynamics regardless of oxygen exposure. Future studies will further evaluate the effects of antenatal inflammation in the presence or absence of different pulmonary vasodilators, such as oxygen and iNO^4,49^ as well as different postnatal injuries, such as hypoxia, hyperoxia, and new infectious insult. As PAAT and RV S:D can be affected by heart rate, hemodynamic conditions, and imaging techniques, we utilized a standard anesthetic approach to ensure that the animals undergo echocardiograms with similar heart rates between 250 and 300 bpm. Furthermore, to account for any variability in heart rate, PAAT was indexed to PAET and reported as PAAT/PAET. We also elected to use RV S:D to assess global RV performance, as the animal size and the RV’s proximity to the sternum prevented us from using tricuspid annular plane systolic excursion, fractional area of change, or deformation imaging. Consistent with previous studies in other rodent models of PH,^50,51^ the appraisal of extrapulmonary shunts and tricuspid regurgitation jet velocity was not feasible in our study. Moreover, measurements of systemic blood pressure and systemic hemodynamics were not included in this study. Another key limitation of this study is the lack of long-term follow-up beyond P14, as pulmonary, pulmonary vascular, and cardiac changes may continue to evolve throughout postnatal development.^6,8^ While our findings provide important insights into neonatal BPD-PH, additional studies will be necessary to determine the later progression of pulmonary vascular remodeling and cardiac adaptation following antenatal ETX exposure. Additional limitations that may restrict the generalizability of our findings include factors such as the altitude of our study site (1600 m), lack of multiple observers, and the quality of echocardiographic images.

In conclusion, we found that impaired lung vascular and alveolar development due to antenatal ETX exposure causes PVD that precedes changes in pulmonary mechanics and the subsequent evolution of echocardiographic metrics of BPD-PH, which increased over time despite the absence of additional postnatal injuries. Our results further strengthen the growing knowledge about mechanisms underlying the development of late PH after preterm birth and the role of antenatal exposures in the pathogenesis of BPD and BPD-PH. We speculate that the early presence of PVD increases the risk for the development of both BPD and BPD-PH and that antenatal exposure to chorioamnionitis is sufficient to increase the susceptibility of premature newborns to develop BPD-PH. Understanding the key role that antenatal factors play in the pathogenesis and progression of BPD-PH is imperative to improve precision in the early identification of at-risk infants and in therapeutic strategies beyond the current BPD approach, which is focused on postnatal therapeutic strategies. Future work should address the effects of antenatal inflammation on the cardiopulmonary structure and function of neonates exposed to prenatal inflammation.

## Data Availability

The datasets generated during and/or analyzed during the current study are available from the corresponding author upon reasonable request.
